# Protein–protein interaction of Rv0148 with Htdy and its predicted role towards drug resistance in *Mycobacterium tuberculosis*

**DOI:** 10.1186/s12866-020-01763-1

**Published:** 2020-04-15

**Authors:** Gunapati Bhargavi, Sameer Hassan, Subramanyam Balaji, Srikanth Prasad Tripathy, Kannan Palaniyandi

**Affiliations:** 1 Department of Immunology, ICMR-National Institute for Research in Tuberculosis, #1, Mayor Sathyamoorthy Road, Chetpet, Chennai, 600031 India; 2 Department of Biological and Environmental Sciences, University of Gothenburg, Gothenburg, Sweden

**Keywords:** *Mycobacterium tuberculosis*, Tuberculosis, Oxidoreductases, Rv0148, Htdy, Drug resistance, Stress response

## Abstract

**Background:**

*Mycobacterium tuberculosis* resides inside host macrophages during infection and adapts to resilient stresses generated by the host immune system. As a response, *M. tuberculosis* codes for short-chain dehydrogenases/reductases (SDRs). These SDRs are nicotinamide adenine dinucleotide-reliant oxidoreductases involved in cell homeostasis. The precise function of oxidoreductases in bacteria especially *M. tuberculosis* were not fully explored. This study aimed to know the detail functional role of one of the oxidoreductase Rv0148 in *M. tuberculosis.*

**Results:**

In silico analysis revealed that Rv0148 interacts with Htdy (Rv3389) and the protein interactions were confirmed using far western blot. Gene knockout mutant of Rv0148 in *M. tuberculosis* was constructed by specialized transduction. Macrophage cell line infection with this knockout mutant showed increased expression of pro-inflammatory cytokines. This knockout mutant is sensitive to oxidative, nitrogen, redox and electron transport inhibitor stress agents. Drug susceptibility testing of the deletion mutant showed resistance to first-line drugs such as streptomycin and ethambutol and second-line aminoglycosides such as amikacin and kanamycin. Based on interactorme analysis for Rv0148 using STRING database, we identified 220 most probable interacting partners for Htdy protein. In the Rv0148 knockout mutants, high expression of *htdy* was observed and we hypothesize that this would have perturbed the interactome thus resulting in drug resistance. Finally, we propose that Rv0148 and Htdy are functionally interconnected and involved in drug resistance and cell homeostasis of *M. tuberculosis.*

**Conclusions:**

Our study suggests that Rv0148 plays a significant role in various functional aspects such as intermediatory metabolism, stress, homeostasis and also in drug resistance.

## Background

Tuberculosis (TB) remains one of the world’s leading cause of death every year among infectious diseases [1]. In 2018, WHO reported that 10.4 million new cases and 1.8 million deaths occur worldwide each year [2]. With the increase in multidrug and extensive drug-resistance TB, there is a need to develop newer diagnostics and vaccines [3]. It is well known that *Mycobacterium tuberculosis* spreads through aerosol, survives in oxygen and nutrition depleted environments and persists for long periods in the host cells [4–6]. The change in the host metabolism is thereby continuously monitored by *M. tuberculosis* to perform its active replication and persistence [7, 8]. *M. tuberculosis* during infection is exposed to various anti-bacterial agents secreted by macrophages. In addition, macrophages produce reactive oxygen species (ROS) and reactive nitrogen species (RNS) [9, 10]. The ROS interact directly and the resulting super oxides are converted into various oxidants like hypochlorite (HClO), peroxides (H_2_O_2_), peroxynitrite (ONOO^*−*^) and hydroxy radicals, which damage the bacterial cells [11]. The bacterial cells resist stress using in-built mechanism. *M. tuberculosis* uses various defence mechanisms to damage the cell. *OxyR* and *soxR* are two prokaryotic regulators against peroxides and superoxides. Due to the absence of *oxyR* in *M. tuberculosis*, peroxidase stress is managed by alkylhydroperoxidase reductase (AhpC) by detoxifying the peroxides [12]. In addition, other peroxidases such as KatG, superoxide dismutase, peroxiredoxin (AhpE) and thioredoxin reductase (Tpx) help in reducing peroxidase activity and also in controlling the oxidative and nitrosative stress conditions [13–15]. Though the components involved in the oxidative stress of *M. tuberculosis* have been identified*,* the functional importance remains unclear. Gene interaction and knockout studies thoroughly predict the functional interconnection between the genes and their role in the metabolism of bacteria.

In this study, for the first time, we attempted to predict the functional role of one of the hypothetical oxidoreductase Rv0148 of *M. tuberculosis*. Rv0148 is a hypothetical protein/putative short-chain dehydrogenase/reductase (SDR) and exhibits 100% similarity to *M. bovis* and *M. africanum* and 87% to *M. avium* [16]. Rv0148 possesses the conserved SDR domain. As aminoglycosides bind to SDR sites, Rv0148 might neutralize the overexpression of aminoglycosides [17]. Earlier studies reported that over expression of *M. tuberculosis* Rv0148 from multidrug-resistance isolates in *Escherichia coli* showed two- to three-folds of higher shift in MIC [18]. Previous studies from our lab identified that PknI, one of the 11 serine-threonine protein kinases (STPKs), interacts with two proteins Rv2159c and Rv0148 [19]. Rv2159c was characterized using gene knockdown studies, and its interaction with PknI increased its peroxidase activity several folds in the mutant strain [20].

As an extension to the previous study we have chosen Rv0148. It was characterized through bioinformatics tools using sequence and protein interaction analyses. In sequence analysis using Pfam database, we found Rv0148 carrying well-conserved nicotinamide adenine dinucleotide (NAD) domain, whereas other homologues possessed MaoC domain along with SDR. Furthermore, by in silico approach we identified that Rv0148 mainly interacts with hydroxyl acyl thioester dehydratase Htdy, a protein interaction which was confirmed by far western blotting (WB) and pull-down assay. We have constructed the gene knock out mutant of Rv0148 (Δ0148) by specialized transduction to understand the functional role of the gene. In vivo studies confirmed that this mutant induces pro-inflammatory cytokines and is susceptible to oxidative and nitrogen stress compounds. Δ0148 confers drug resistance to streptomycin, ethambutol, amikacin and kanamycin. This might be due to modified functional network in the absence of Rv0148. Overall, the study suggested that Rv0148, though a non-essential gene, is functionally involved in drug resistance and is interconnected with other drug-resistance genes. The absence of Rv0148 displays its prominent role in host immunity.

## Results

### Sequence homologues of Rv0148

**Fig. 1:**
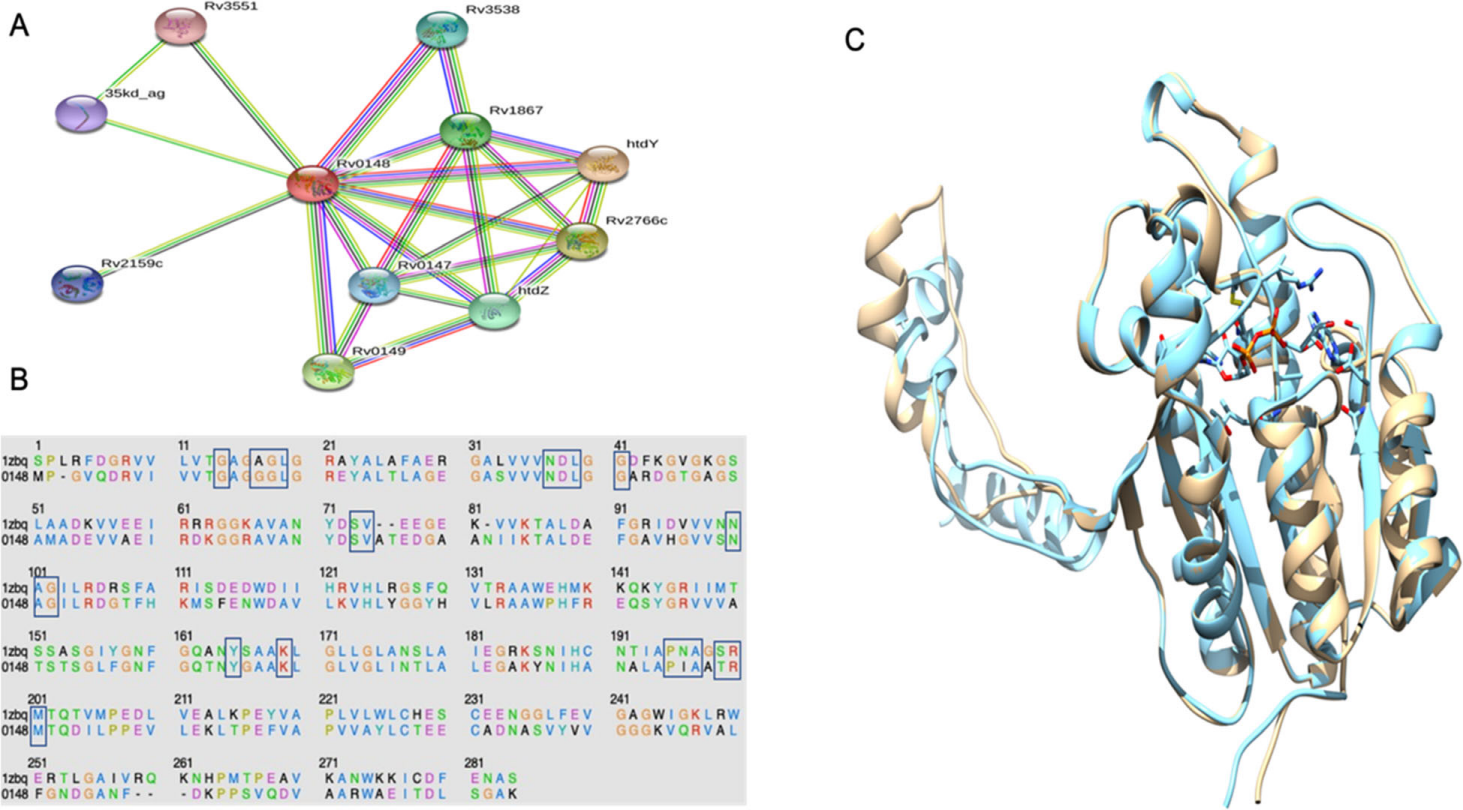
**a** Protein-protein interaction from the STRING database for Rv0148 protein. **b** Sequence comparison of Rv0148 with a template sequence (1ZBQ). The black boxes are the mapping binding sites for Rv0148 based on the template sequence. **c** The structural superimposition between nicotinamide adenine dinucleotide (NAD)-docked RV0148 (brown) and template, 1ZBQ (cyan). The NAD is represented in stick form

To identify the sequence homologues of Rv0148 in other organisms, blast search was performed against the non-reductant database in NCBI; 104 sequence homologues were selected and the sequences were reconfirmed based on short dehydrogenase domain in these sequences. Most of these sequences had the short dehydrogenase with a few sequences having both short dehydrogenase and MaoC dehydratase domain. A HMM profile based analysis (https://www.ebi.ac.uk/Tools/hmmer/search/hmmsearch) using the adh short domain profile from Pfam database identified 1270 significant sequences from Swissprot database (Table S1). Further, we searched the MaoC dehydratase sequence against mycobacterial genome and identified Htdy protein. Based on the analysis, we identified Rv0148 has only SDR. We searched for Rv0148 in STRING database and identified that Rv0148 interacts with Htdy protein (Fig. 1a). And this formed the basis for the subsequent analysis to predict the interaction of Rv0148 with Hdty using bioinformatics tools and then to validate the interaction using experimental methods.

### Homology modelling of Rv0148

**Fig. 2:**
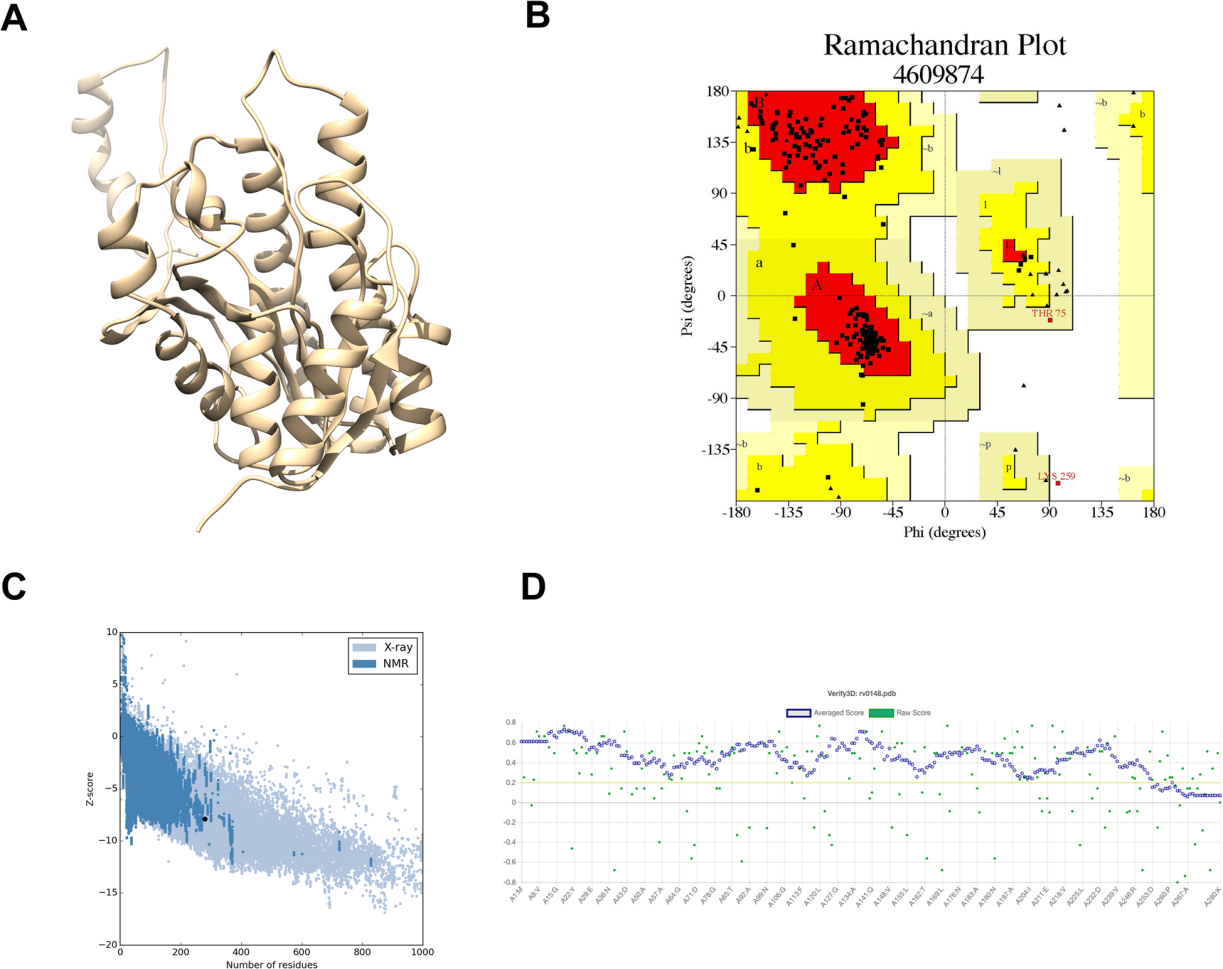
**a** Predicted 3D structure of Rv0148. **b** Ramachandran plot for the predicted model structure of Rv0148. **c** Plot showing residue energy with *Z*-score for the Rv0148 model. The blue and grey colours represent the experimentally determined structures using NMR and X-ray, respectively. The *Z*-score for Rv0148 model falls within the range of the experimentally determined structures. **d** Plot showing the average 3D-1D score for every residue of the model structure

Because the structure of Rv0148 was not determined by experimental methods, we attempted to predict its 3D structure using the homology modelling approach. From the blast search results against PDB for a suitable template in complex with NAD, it was observed that Rv0148 showed the best alignment with human 17-beta-hydroxysteroid dehydrogenase type 4 in complex with NAD (PDB code 1ZBQ) and was chosen as the template. The sequence coverage between Rv0148 and 1ZBQ was 95% with a sequence identity of 52%, *E*-value of 2e-96) and gap of 2%. Both Rv0148 and 1ZBQ have short-chain dehydrogenase domain (PF00106). The 3D structure of Rv0148 was modelled (Fig. 2a) using the model-default python script in MODELLER software and the sequence alignment used for predicting the model structure is shown in Fig. 1b. The predicted model was validated using PROCHECK, profile-3D, and ProSA-web. Stereochemical properties of the predicted model were evaluated using PROCHECK to analyse the Ramachandran plot (Fig. 2b).

The plot revealed that the occurrence of 92.3% amino acids was in the favoured region, 7.3% in the generously allowed region and 0.4% in the disallowed region. *Z*-score was − 7.89 for the model structure determined using ProSA-web, a program used for validating protein structure, and was located within the space determined by X-ray crystallography (Fig. 2c). Thus, the overall results from structural superimposition, Ramachandran plot, verify-3D, and ProSA-web reveal that the predicted model is satisfactory and thus considered for further analysis. Profile-3D of the Rv1048 model was 90.36, thus suggesting that 90.36% of the residues have an average 3D–1D score ≥ 2 (Fig. 2d).

### Docking of NAD within the active site of Rv0148

The docking of NAD co-factor was performed using GOLD software. However, a primary docking of the bound NAD to the template structure 1ZBQ was re-docked in its binding site to evaluate the docking process. The RMSD of the resulting docking of NAD to 1ZBQ was 0.86 Å, suggesting that the docking program is able to reproduce a structure very close to the experimental structure. In order to discover the putative binding pose of NAD co-factor in Rv0148, the binding site for Rv0148 was mapped based on 1ZBQ (Fig. 1b). The best-predicted pose of NAD within the active site of Rv0148 had a score of 95.44 Å (Fig. 1c).

### Rv0148 and Htdy interaction analysis

**Fig. 3:**
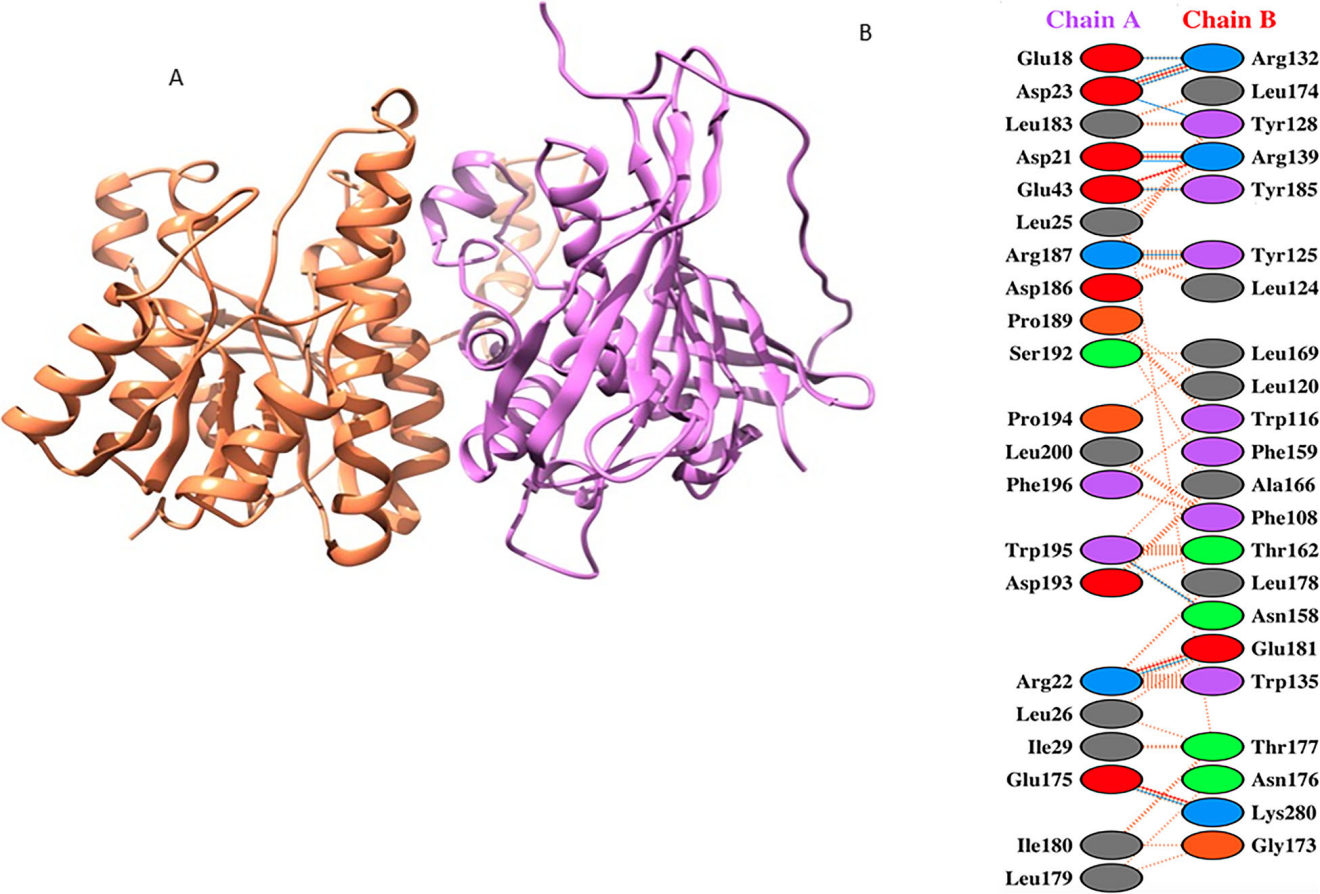
**a** The predicted interaction of Rv0148 (orange) and Htdy (purple) protein structures using ClusPro protein-protein interaction server. **b** The figure showing interacting residues between the two proteins. Chain A & B, chain A represents Htdy and chain B represents Rv0148

The crystallographically determined structure of Htdy (PDB code 3KHP) and the predicted model structure of Rv0148 were used for docking using the ClusPro server. The structure from the largest cluster and with the lowest energy was selected and examined for the interaction between the two complexes (Fig. 3a). The residues interacting between the two proteins Rv0148 and Htdy from the docked complex were then mapped to identify the active site (Fig. 3b).

### Cloning, expression and purification of Rv0148 and Htdy

The coding region of the two genes was amplified by PCR and the product matches the size of Rv0148 with 858 bp and *htdy* with 969 bp. The *E. coli* strain (BL21) expressed pGBRJ3 Htdy as His-tagged protein and pGBRJ2 0148 as GST-tagged protein (Fig. S1). The protein was purified. The mass of Htdy is 31 kDa and Rv0148 GST is 55 kDa. These correspond to the expected mass of the above proteins through western blot.

### Interaction of Rv0148 and Htdy

**Fig. 4:**
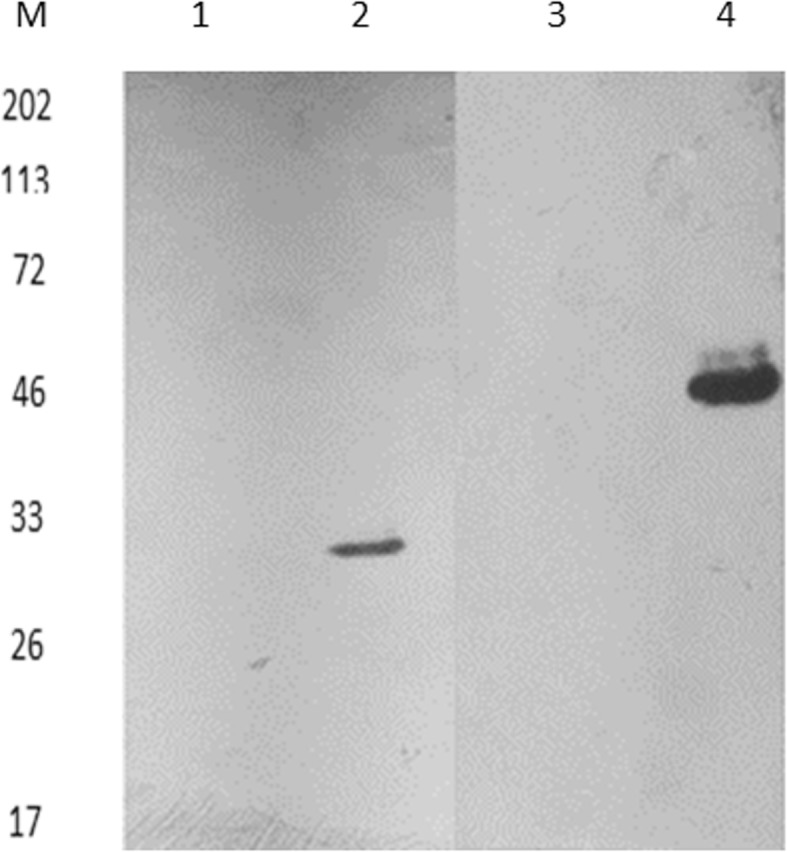
Rv0148 interacts with Htdy. Pull-down assay confirms the interaction between Rv0148 and Htdy. The crude lysates enclosing His-Htdy and GST-Rv0148 were incubated with Ni-NTA resin; unbound proteins (lane 1) were washed off and incubated with their corresponding substrates. The protein fractions were eluted (lanes 2 and 4) and immunoblotted with anti-His and anti-GST antibodies. As a control, the bait proteins were incubated with bovine serum albumin protein (lane 3). Lane M, molecular mass standard

The pull-down assay with recombinant His-Htdy (PGBRJ3) lysate using Ni-NTA affinity chromatography confirmed that the Htdy was able to pull down GST-tagged Rv0148 (PGBRJ2). Simultaneously, the GST-tagged lysate was able to pull down His tag recombinant Htdy (Fig. 4). The pull-down assay and far western blot confirmed that Rv0148 interacts with Htdy protein.

### Generation of ∆0148 knockout mutant in *M. tuberculosis*

**Fig. 5:**
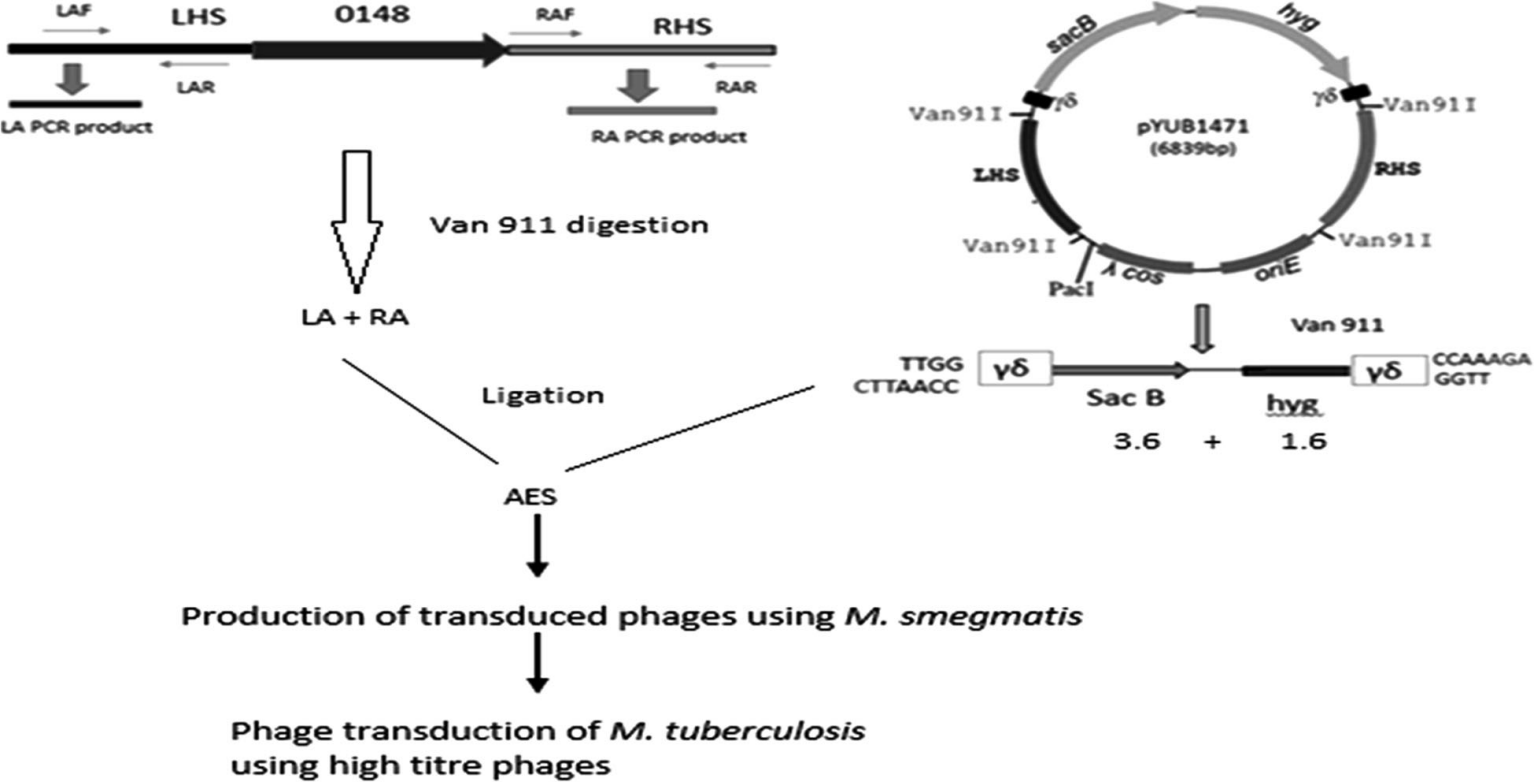
Overview of gene knockout. Pictorial representation of steps followed for successful construction of gene knockout mutant

We constructed knockout mutant of *M. tuberculosis* for not expressing Rv0148 by phage-facilitated allelic exchange [21]. The coding region of the gene was replaced by the hygromycin cassette and four fragments were generated with 3.6, 1.6, 0.837 and 0.605 bp product sizes (Fig. S1). The 50 kb phAE159 shuttle phasmid with AES retains 6.642 kb **(**Fig. S2**).** The generated high-titre transducing phages were used for specialized transduction and the knockout mutant was generated successfully and named as Δ0148 (Fig. 5). We confirmed the knockout mutant using sequencing, PCR for hygromycin cassette and also through real-time PCR (RT-PCR) using gene-specific primers (Table S3).

### Colony morphology and growth kinetics

Gene disruption shows no distinct effect in the cell development of the knockout strain compared to H_37_Rv. To elucidate if the knockout of Rv0148 confers any change in vitro, we compared the growth kinetics of H_37_Rv, ∆0148 and CΔ0148 in 7H9-enriched media. All the strains showed similar growth and there was no distinct difference observed in in vitro survival of ∆0148.

### Cytokines analysis using ELISA

**Fig. 6:**
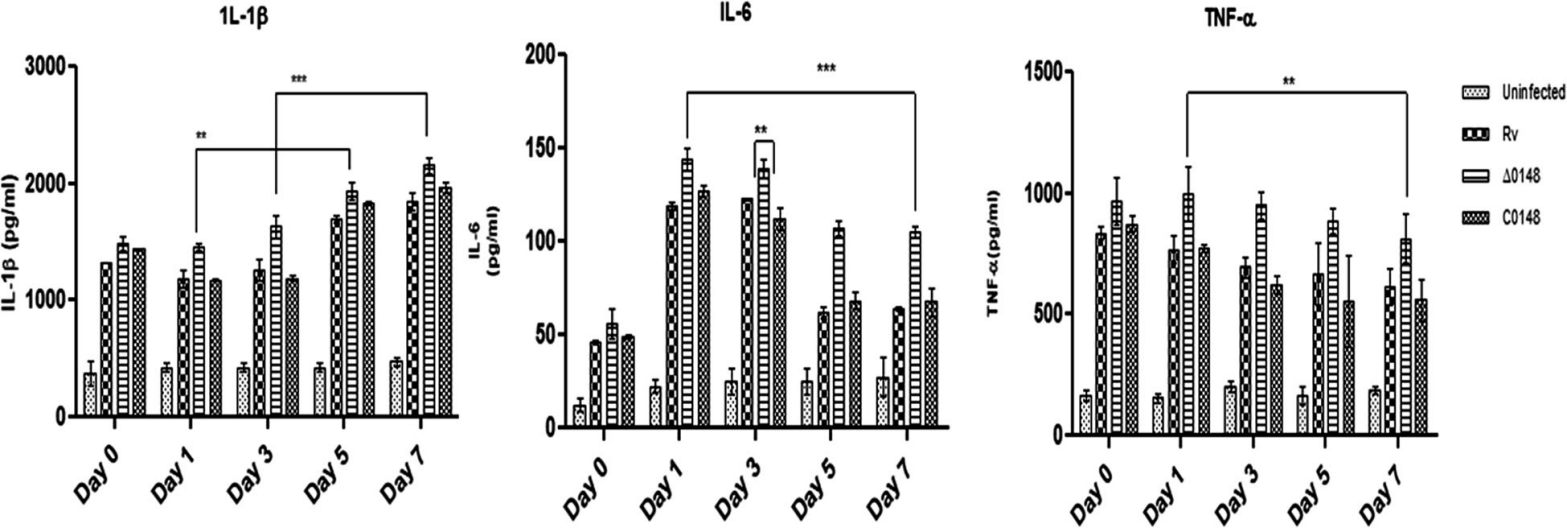
Cytokines secreted by macrophages infection. H_37_Rv, Δ0148 and CΔ0148 strains after post-infection cytokines were measured according to protocol. The capture antibody was coated onto the wells; to samples of 100 μL were added detection antibody followed by substrate solution to identify the specific cytokines. IL-1β, IL-6 and TNF-α. Two-way ANOVA was performed. The values were taken by considering the average of three triplicate independent experiments. Data represents mean, Standard error mean deviation (SEM) in each experiment and error bars indicate mean ± standard deviation SD. *** significant at *P* < 0.001 and ** significant at *P* < 0.01

The cell-free supernatants after post-infection were analysed for the expression of pro-inflammatory cytokines IL-1β, IL-6 and TNF-α and anti-inflammatory cytokines IL-10, IL-12p40, IL-12p70 and IL-4. Enhanced expression of IL-1β, IL-6 and TNF-α cytokines was observed in ∆0148 infected with THP-1 macrophages (Fig. 6a–c). Similarly, reduced level of expression of IL-10, IL-12p40, IL-12p70 and IL-4 was observed in ∆0148 compared to wild-type H_37_Rv (data not shown). The expression was analysed using appropriate statistical methods.

### Effect on inhibition of the electron transport chain in ∆0148

**Fig. 7:**
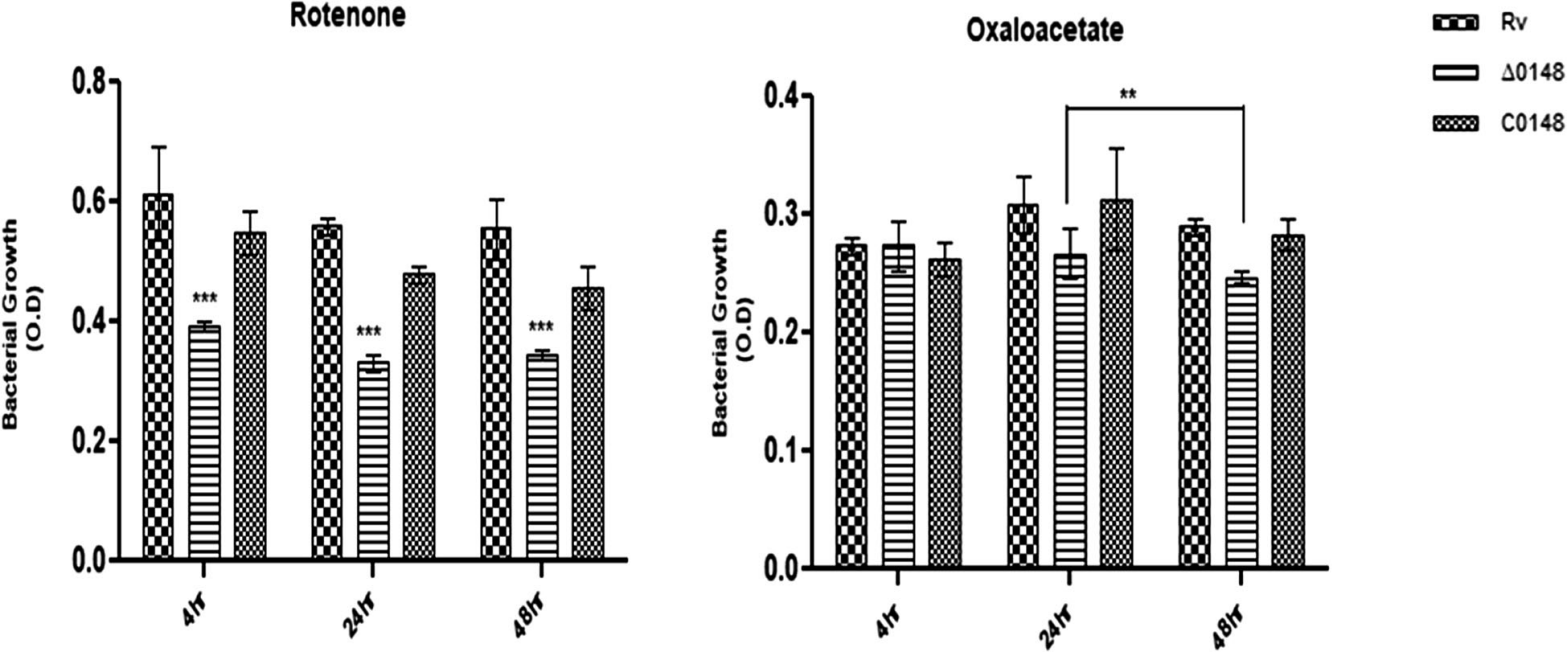
Effect of electron chain inhibitors. H_37_Rv, Δ0148 and CΔ0148 strains were grown to mid-log phase and the culture diluted with 7H9 broth. The OD600 was measured after post-infection time points of 4, 24 and 48 h with 140 μM rotenone and 2 mM oxaloacetate. The represented data is the mean ± SD of three independent experiments. Two-Way ANOVA, standard error mean was performed. *** significant at *P* < 0.001 and ** significant at *P* < 0.01

Rv0148 is predicted to be involved in the intermediatory metabolism of *M. tuberculosis.* The ETC plays an important role in the production of proton motive force (PMF) in the metabolism of bacteria. ETC consists of five complexes I, II, III, IV and V, and inhibition of these complexes could accommodate changes in the growth of bacteria. Rotenone is an inhibitor of complex I whereas oxaloacetate inhibits complex II. It was observed that the exposure of Δ0148 to 140 μM rotenone and 2 mM oxaloacetate significantly decreases the growth (Fig. 7a and b)**.** The result suggests that ∆0148 strain has a distinct role in the ETC. The significance was reported using statistical tools.

### Effect of oxidative and nitrosative stress in ∆0148

**Fig. 8:**
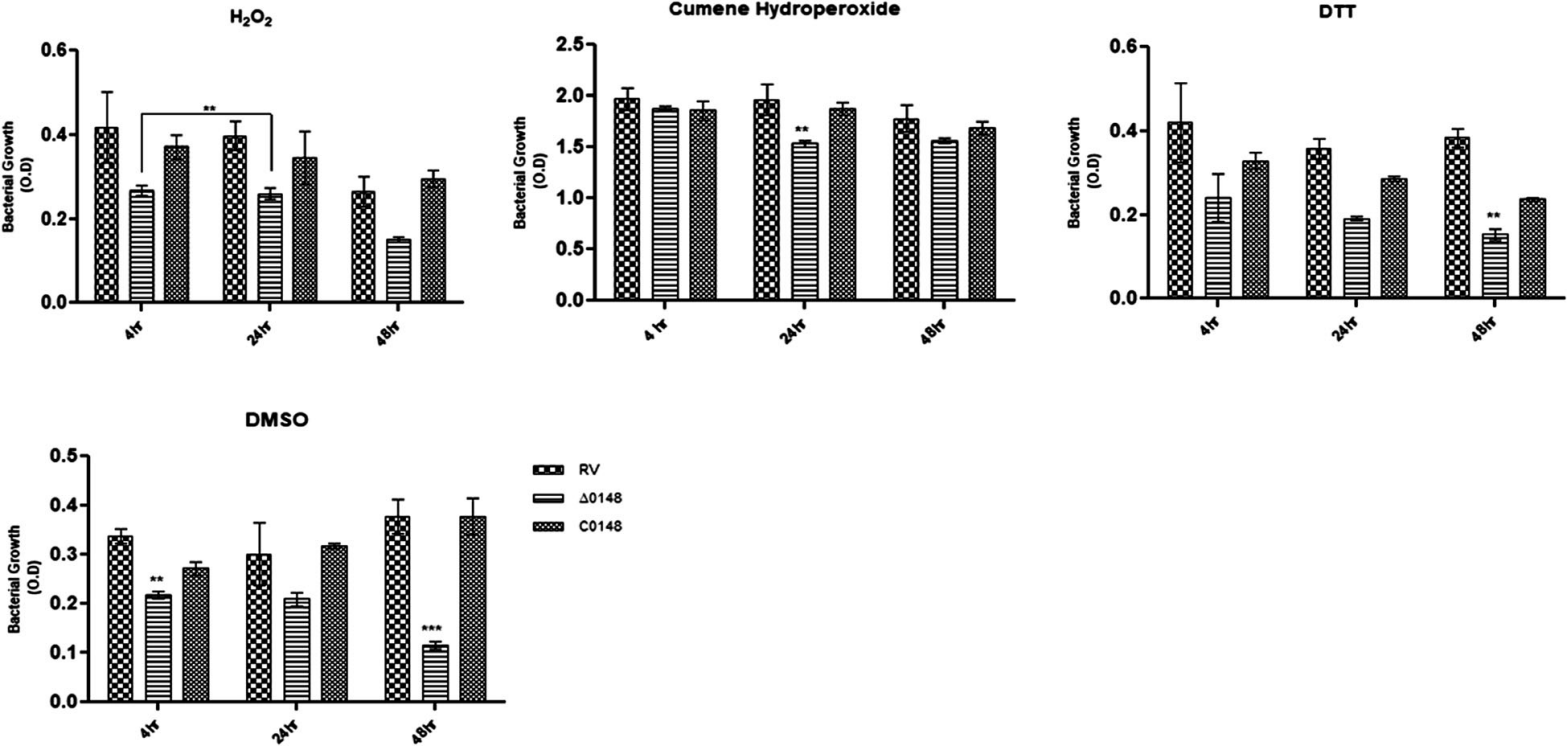
Effect of stress in Δ0148. The mid-log phase cultures were subjected to oxidative and nitrogen stress compounds and OD values were measured after post-exposure with 5 mM H_2_O_2_, 5 mM cumene hydroperoxide, 2 mM dithiothreitol and 1% DMSO at 4, 24 and 48 h. The values were taken by performing three independent experiments. The bar graphs were plotted by considering the mean, SEM and using Two-Way ANOVA and error bars indicate mean ± SD. significant at *P* < 0.001 and ** significant at *P* < 0.01

In diseased patients, mycobacterium resides in the alveolar macrophages in the initial stage of infection and secretes various ROS and RNS. We investigated whether these stresses affect the metabolism of ∆0148 strain when exposed to 5 mM hydrogen peroxide (H_2_O_2_), 5 mM cumene hydroperoxide, 2 mM thiol-reductant DTT and 1% sulphoxide (DMSO). ∆0148 is sensitive to oxidative stress components and to nitrogen derivatives and there is decrease in growth of Δ0148 compared to wild-type H_37_Rv (Fig. 8a–d). It was concluded that the growth of ∆0148 declined on exposure to ETC, oxidative and nitrosative stress agents. The result was considered using standard statistical methods.

### Sensitivity and resistance to first-line and second-line drugs

BACTEC MGIT was performed with first-line and second-line drugs and shows that ∆0148 is resistant to streptomycin, ethambutol, amikacin, and kanamycin. H_37_Rv was maintained as a control for all these drug sensitivity assays (Table 1).

**Table 1:** Resistance and sensitivity to drugs

Name of sample	Strep (1 μg/mL)	INH (0.1 μg/mL)	Eth (5 μg/mL)	RIF (1 μg/mlL	KAN (2.5 μg/mL)	OFLO (2 μg/mL)	AMI (1 μg/mL)
H_37_Rv	S	S	S	S	S	S	S
Δ0148	R	S	R	S	R	S	R

Abbreviations: *AMI* amikacin, *Eth* ethambutol, *INH* isoniazid, *KAN* kanamycin, *OFLO* ofloxacin, *R* resistance, *RIF* rifampicin, *S* sensitive, *Strep* streptomycin

### Interactomic analysis

**Fig. 9:**
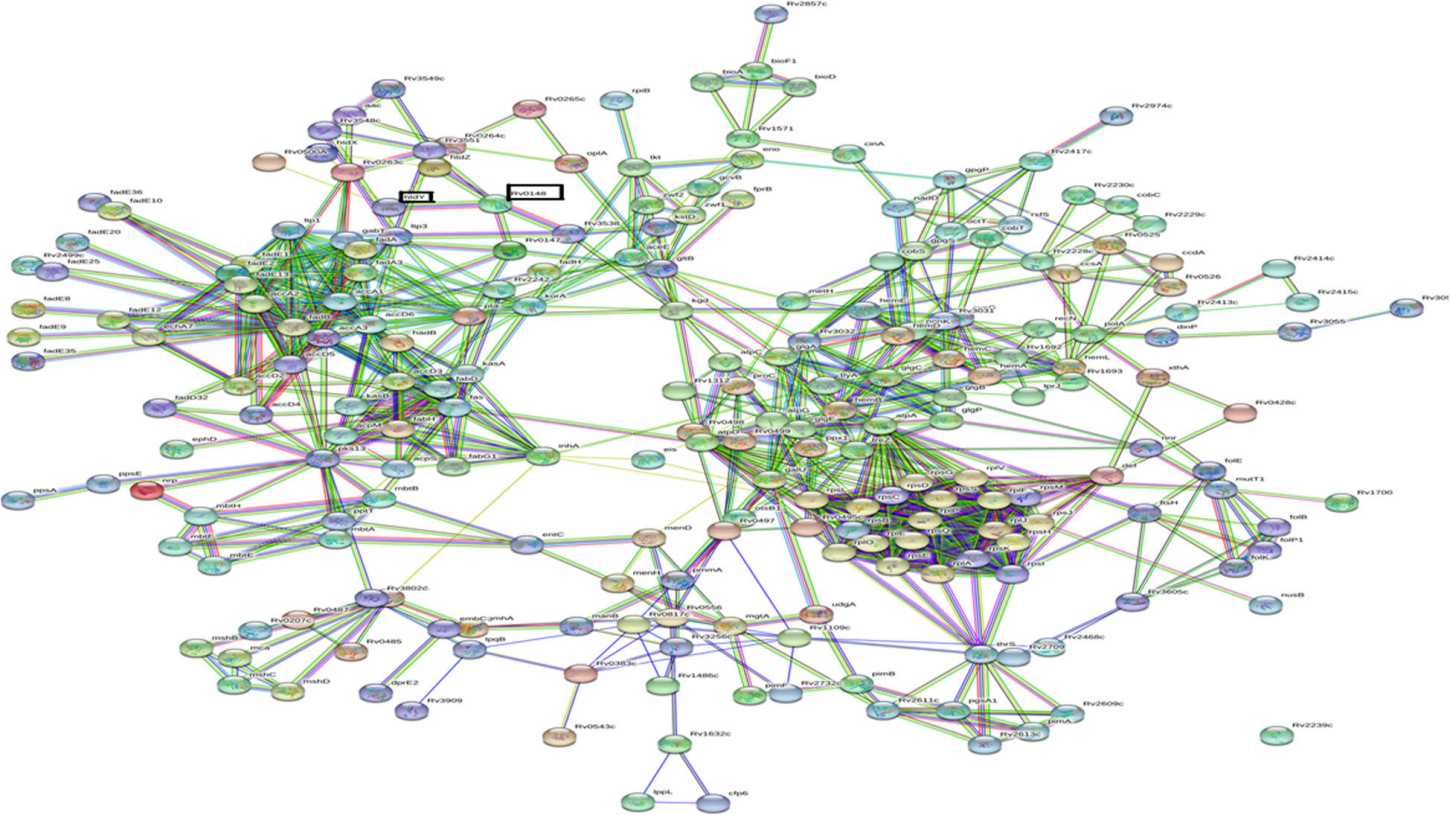
Interactome showing 220 genes interacting with Rv0148

The STITCH database was used to identify *M. tuberculosis* proteins that interact with three anti-TB drugs streptomycin, kanamycin and amikacin. For each of the proteins interacting to the respective drugs identified from the STITCH database, we collected the interactomes with high confidence > 0.7 from STRING database to ensure a level of confidence for the interactions. Simultaneously, we collected the highest confidence interactomes for Rv0148, *htdy* and EIS genes from STRING (Table 2). Finally, we produced an interactome of 220 proteins that connect Rv0148, Htdy and EIS with the proteins that either interact directly or indirectly with the three drugs. The *M. tuberculosis* PPI (protein–protein interaction) network incorporating all the identified proteins is shown in Fig. 9. Comparing and analysing the intersection of proteins that connect the three drugs kanamycin, amikacin and streptomycin and the three proteins Rv0148, Htdy and EIS resulted in 57 proteins (Fig. S3). Analysing the 220 genes, except for streptomycin, we identified genes connected to kanamycin and amikacin as an intersection between interacting genes of Rv0148, *htdy* and EIS.

**Table 2:** List of drug-resistance genes interacting with Rv0148

Rv0148 vs kanamycin	Rv0148 vs EIS	EIS vs amikacin	Rv0148 vs Htdy
			fadb
Rv3538	accA2	eis	accA2
	Fas	Rv2417c	Rv3538
Fas	kasA	Rv2414c	Rv0148
Htdy	fabD	Rv3055	fas
Htdx	kasB	Rv1312	acps
Rv0130	AccD3	Rv1571	kasA
mutT1	eis	Rv3605c	Pks13
		Rv3839	mbtB
		Rv0428c	Rv0216
		Rv0495c	Rv2499c
		Rv2242	Htdx
			hatB
			fabD
			Rv0130
			Rv3797
			FadE9
			fadE25
			fadE10
			fadE8
			fadE20
			FadE12
			fadA
			FadA3
			Rv0147
			accD4
			Rv3548c
			kasB
			Rv2228c
			mutT1
			accD2
			echA7
			accD3
			fadE13

### Differential expression of *htdy*

**Fig. 10:**
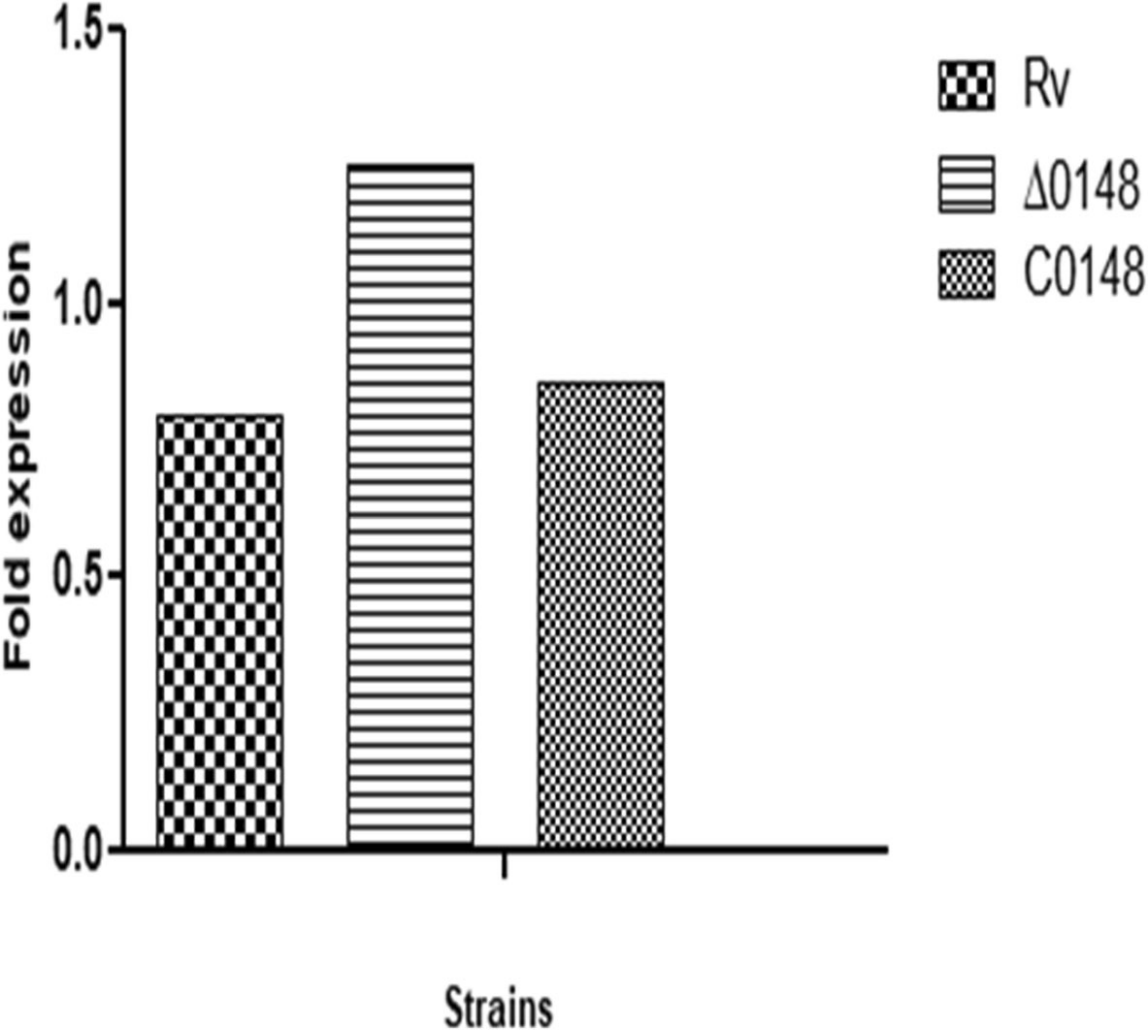
Expression of *htdy* in Δ0148 strain. The bar graphs represent the fold expression of *htdy* in H_37_Rv, Δ0148 and C0148. The expression were analysed using SDS software and relative quantity method

The interactome analysis predicted that *htdy* is also involved in drug resistance. Expression of *htdy* in the Δ0148 strain of *M. tuberculosis* was analysed using qPCR as mentioned earlier. The *htdy* expression in Δ0148 increased to 1.25-fold whereas the wild-type strain showed 0.7-fold (Fig. 10 and Table S4). The fold expression was calculated using SDS 2.4 software and relative quantity (RQ value) method.

## Discussion

In this study, we report the cloning, overexpression, purification, gene knockout and interacting partners of Rv0148, a hypothetical protein that belongs to the oxidoreductase family of *M. tuberculosis*. Through bio-informatics approaches, we concluded that Rv0148 is a NAD oxidoreductase protein with SDR domain. Rv0148 is predicted as interacting with a protein that has an MaoC dehydratase domain and the subsequent blast analysis led to the identification of Htdy protein. Further, the string analysis of Rv0148 displayed 10 interacting partners such as Rv3538, Rv0149, *mbtB*, *fas*, *fadB*, *aacA2*, Rv0147, Rv2228c, *muT1* and *htdy* thus confirming our analysis. The predicted Htdy as an interacting partner of Rv0148 was further confirmed through far WB and pull-down assays.

We generated gene knockout mutant of Rv0148 in *M. tuberculosis* and show that Rv0148 is a non-essential gene as confirmed in the previous report [22]. ∆0148 grew similarly as the wild type under constant shaking in enriched 7H9 media. This indicates clearly that the loss of Rv0148 does not lead to any change in the growth of *M. tuberculosis*. There were no correlative morphological changes in cell structure owing to the deletion of Rv0148.

We infected the macrophage cell lines with mutants for cytokine profiling. Increased expression of pro-inflammatory cytokines predicts that Δ0148 might be involved in host immune responses [23, 24]. The lower level expression of anti-inflammatory cytokines IL-10, IL-4, IL-12p40 and IL-12p70 indicates that Δ0148 has no role in inflammation [25].

In aerobic organisms, respiratory chain possesses inner membrane complexes associated with energy production. Complex I known as NADH quinone oxidoreductase transfers electrons from NADH to quinone associated with proton transfer across the membrane. Complex II is involved in tricarboxylic acid cycle without transfer of protons. Complex III transfers electrons from quinone to cytochrome *c*. Complexes III and IV receive these electrons and transfer them to oxygen. Complex V using PMF synthesizes ATP molecules [26]. Rv0148 is a NAD oxidoreductase involved in the function of intermediary metabolism and respiration. The exposure of Δ0148 to components inhibiting ETC complexes I and II [27] revealed that ∆0148 responds to changes in the electron transport system, thus predicting its role in cell homeostasis. *M. tuberculosis* adapts to various stress conditions to survive as a successful pathogen inside the host [28]. Δ0148 interestingly showed decreased growth in hydrogen peroxide, CHP, DTT and DMSO, which indicates its sensitivity to oxidative and nitrogen stress. Thus, in response to stress, the differential growth of ∆0148 demonstrates that the gene plays an important role in the protective efficacy against oxidative and nitrogen stress [29].

Contradictory to previous findings, Rv0148 showed increased expression in MDR isolates [18]. In the present study, Δ0148 showed resistance to first-line streptomycin and ethambutol and second-line amikacin and kanamycin drugs and the result was correlated with its interaction towards drug-resistance genes. Interactome analysis in the study suggests that Rv0148 interacts with kanamycin resistance genes Rv3538, *mutT1*, *fas*, Rv0130, Rv3389 and *htdx*. The EIS gene is reported earlier to be involved in causing drug resistance of *M. tuberculosis* [30]. Our analysis reveals that Rv0148 interacts with *eis* genes *accA2*, *fas*, *kasA*, *fabD*, *KasB* and *accD3*. Rv0148 does not display any direct interaction with amikacin and ethambutol genes; however, the interactomes of EIS and amikacin share common interacting genes such as Rv2417c, Rv2414c, Rv3055, Rv1312, Rv1571, Rv3605c, Rv3839, Rv0428c, Rv0495c and Rv2242. As these proteins are connected to each other indirectly on a larger interactome space, we assume that drug resistance in Δ0148 strain could be due to some disruption of the interactome network.

The major findings from the current study prove that *htdy* is also involved in drug resistance and the increased expression of *htdy* in ∆0148 predicts that Rv0148 altered the expression of interacting partners in *M. tuberculosis*. Interactome analysis using STRING database predicts that Rv0148 has a strong interaction with kanamycin, *eis* and amikacin resistance genes. Further, interactome analysis reveals that Rv0148 and Htdy are connected to EIS and the interacting proteins of the drugs analysed in this study. Thus, we assumed that the absence of Rv0148 affects the interactome that might bring a major expression difference in these proteins resulting in drug resistance towards respective drugs. An additional transcriptome profiling of these strains will further help in identifying a clear mechanism towards drug resistance. Further, studies like double knockout mutant of Rv0148 and Rv3389 will provide the detailed functional role of these genes.

## Conclusion

To summarize, Rv0148 is a non-essential gene belonging to the oxidoreductase family in *M. tuberculosis*. In silico approach and protein interaction studies reveal that Rv0148 interacts with Htdy, which is a protein of the hydroxyl acyl family. Htdy in the presence of Rv0148 is quite stable and fold expression of the gene is optimal. In the absence of Rv0148, increased fold expression of *htdy* proves that these two genes are functionally interconnected and furthermore it is experimentally proved. In addition to these findings, Rv0148 not only interacts with *htdy*, but also has a strong interactome network of 220 genes in *M. tuberculosis* with a high confidence of 0.7. The absence of Rv0148 showed resistance to first-line drugs streptomycin and ethambutol and second-line drugs amikacin and kanamycin. Increased expression of pro-inflammatory cytokines suggests that Δ0148 might play a role in targeting the immune response. Our study suggests that Rv0148 plays a significant role in various functional aspects such as intermediatory metabolism, stress and homeostasis.

## Methods

### Template selection and homology modelling

A suitable template for Rv0148 was identified by searching in the PDB database using the default settings of BLAST program in February, 2017. The sequence of Rv0148 was searched against Pfam database to further identify available PDB structures having the same domain. The final template (PDB Id: 1ZBQ) was chosen based on sequences having the highest sequence identity and domain coverage against Rv0148 sequence. The template 1ZBQ selected for modelling Rv0148, is the experimentally determined crystal structure of human 17-beta-hydroxysteroid dehydrogenase in complex with cofactor NAD. The template and the target sequence were aligned using the malign program in Modeller software [31]. Homology model of Rv0148 was constructed using Modeller and the quality of the model was further analysed using PROCHECK [32], ProSA-web [33] and verify 3D [34]. The model was also validated using root mean square deviation (RMSD).

### Molecular docking

Docking was performed using NAD cofactor as the ligand with the predicted model of Rv0148 to understand its mode of interaction within the binding site. GOLD v5.2 (genetic optimization for ligand docking) is a widely used protein–ligand docking program [34]. GOLD uses a genetic algorithm methodology for protein–ligand docking that allows full ligand and partial protein flexibility. The homology model of Rv0148 protein was used as the receptor after the addition of hydrogen atoms before docking. The NAD ligand was corrected with Auto Edit Ligand option of GOLD program. Default genetic algorithm (GA) settings that ensure 100% search efficiency were used for docking. A total of 20 poses of the docked molecule were generated. Early termination of the number of GA runs was allowed when the RMSDs of the top three GA solutions were within 1.5 Å. The best pose of the docked ligand was selected based on Goldscore.

### Protein–protein docking

The interactions between Rv0148 and Htdy structures were docked using ClusPro [35]. The ClusPro algorithm uses fast fourier transform correlation, making it feasible to generate billions of docked conformations by simple scoring functions. ClusPro filters the docked confirmations with near-native structures, ranks them based on their clustering properties and finally refines a limited number of structures by energy minimization. The server outputs the top predicted complexes based on energy and cluster size.

### Bacterial strains and growth conditions

*E. coli* strains DH5α and BL21 (DE3) (Invitrogen, USA) were used for cloning and expression of recombinant proteins. *E. coli* cells were grown in Luria Bertani (LB) medium with constant shaking at 37 °C. *M. smegmatis* (mc^2^155) was grown in Middlebrook 7H9 broth (Difco, USA) with 0.2% glycerol and 0.05% Tween-80. The media was supplemented with 50 μg/mL ampicillin for *E. coli* whenever needed. *M. tuberculosis* was also grown in 7H9 broth with antibiotics carbenicillin 50 μg mL, cyclohexamide 10 μg mL, kanamycin 25 μg mL and hygromycin 50 μg mL, wherever required, along with OADC and Tween-80*.* Primers used in the study are listed in Table S2. The plasmids and strains constructed in the present study are mentioned in Table 3.

**Table 3:** Plasmids and bacterial strains constructed in the study

Plasmid	Source of description	Reference/origin
*E. coli* DH5α	F^−^*end*A1 *gln*V44 *thi-1 rec*A1 *rel*A1 *gyr*A96 *deo*R *nup*G ϕ80d*lacZΔ*M15 Δ(*lacZYA-argF*)U169, *hsd*R17(rK-mK+), λ-	Invitrogen
*E. coli* BL21 (DE3)	F^−^*ompT gal dcm lon hsdS*_*B*_*(r*_*B*_^*−*^*m*_*B*_^*−*^) λ(DE3 [*lac*I *lac*UV5-T7 *gene* 1 *ind*1 *sam7 nin5*]	Invitrogen
*M. smegmatis mc*^*2*^*155*	Lab stock	
pGEX 4 T-1	lacT^q^, 4.9 kb, Amp^r^, GST gene fusion vector	GE Healthcare
pRSET-B	lacT^q^, 2.8 kb, Amp^R^, 6xHis (N-term) gene fusion vector	Invitrogen
p0004- Sac B	Suicide recombination delivery vector carrying *hyg*^*R*^-*SacB* for gene disruption, hyg^R^	
PhAE159	Conditionally replicating shuttle phasmid vector	*William R Jacobs Jr* (unpublished)
pMV261	*E. coli* mycobacterial shuttle vector, Kan^R^, *hsp*60 promoter	Strover et al. (1991)
Constructs in this study		
pGBRJ1	pRSET-B vector carrying the coding region of Rv0148 gene from *M. tuberculosis*, Amp^R^	This study
pGBRJ2	pGEX 4 T-1 vector carrying the coding region of Rv0148 gene from *M. tuberculosis*, Amp^R^	This study
pGBRJ3	pRSET-B vector carrying the coding region of *Htdy* gene from *M. tuberculosis*, Amp^R^	This study
Δ0148	The deletion of Rv0148 gene from *M. tuberculosis*	This study
CΔ0148	pMV261 vector carrying the coding region of Rv0148 gene from *M. tuberculosis,* Kan^R^ expressed in *M. tuberculosis* (complementation)	This study

### Cloning and expression of Rv0148 and *htdy*

*M. tuberculosis* genomic DNA was isolated using CTAB-NaCl standard method [36]. Coding region of Rv0148 from *M. tuberculosis* was amplified by polymerase chain reaction (PCR) using gene-specific oligonucleotides. The PCR product of *htdy* was further cloned into pRSET-B harbouring *T7* promoter and Rv0148 in pGEX 4 T-1 vector harbouring *tac* promoter with Bam HI at 5′ and Xho I at 3′ end as restriction sites. The resultant clone was confirmed through restriction digestion and DNA sequencing. For recombinant protein expression, pGBRJ3 and pGBRJ2 clones were transformed into *E. coli* BL21 (DE3) cells and protein expression was induced with 0.5 mM isopropyl β-d-1 thiogalactopyranoside (Invitrogen, USA). Induced cells were grown for 4 h at 37 °C and harvested by centrifugation at 8000 rpm for 10 min. The cell pellet was re-suspended in ice-cold lysis buffer G (50 mM Tris pH 8, 300 mM NaCl, 20% glycerol,0.5 mM phenyl methyl sulphonyl fluoride and 10 mM imidazole) along with protease inhibitor cocktail Sigma fast (SIGMA, USA) and sonicated on ice using ultrasonic homogenizer (5 cycles: 30 s pulse and 1 min interval). Finally, the lysate was centrifuged at 10,000 rpm for 10 min at 4 °C to separate soluble and insoluble proteins.

### Purification of Rv0148 and Htdy

The soluble lysate proteins were analysed on 12% SDS gel for confirmation of recombinant proteins and protein lysis. Lysate was purified using affinity column (Bio-RAD, CA, USA) and 6X histidine-tagged protein was bound to NI-NTA resin (Invitrogen, USA) and incubated at 12 °C for 2 h. The column was washed with 10 column volumes of wash buffer containing 40 mM imidazole and was eluted with a buffer containing 200 mM imidazole. Similarly, GST-tagged protein was bound to glutathione sepharose resin (GST, Takara, Japan) and eluted with 20 mM reduced glutathione. The eluted fractions were then dialysed and analysed by SDS-PAGE and WB.

### Pull-down assay

Pull-down assay was performed using poly-prep chromatography column (Bio-Rad, USA) with NI-NTA resin. Recombinant pGBRJ3 His lysate was incubated with NI-NTA resin for 2 h at 12 °C with gentle agitation. The column was washed with buffer containing 40 mM imidazole and incubated overnight with pGBRJ2 GST lysate from *E. coli* BL21 (DE3) cells at 12 °C with gentle agitation at 10 rpm in an end-to-end shaker. Unbound proteins were removed by washing with 10 column volumes of buffer. The interacting proteins were eluted with buffer containing 200 mM imidazole.

### Far western blot

To recognize the Rv0148 interacting proteins, the fraction eluted in pull-down assay was separated on a 12% SDS-PAGE and transferred to Immobilon-P (PVDF) membrane. The membrane was denatured by incubation with varying concentrations (6, 3, 1 and 0.1 M) of guanidine-HCl in AC buffer containing 5 M NaCl, 1 M Tris pH 7.5, 0.5 M EDTA, 10% Tween-20, 2% skimmed milk powder, 10% glycerol and 1 M dithiothreitol (DTT) for 30 min at room temperature followed by 5 min wash with phosphate buffer saline Tween 20 [37]. Then, the blot was completely renatured in AC buffer without guanidine-HCl and left overnight at 8 °C. The membrane was then blocked with 5% skimmed milk and incubated with bait protein for 7–8 h at 8 °C and further with 1:6000 anti-His monoclonal antibody and developed using enhanced chemiluminescent kit (Thermo Scientific, USA).

### Construction of allelic exchange substrate

Gene disruption was carried out using Rv0148 left homology sequence (LHS) and right homology sequence (RHS) flanking regions carrying van911 as restriction site targeted for deletion and substitution. The gene was cloned onto either side of the selectable antibiotic cassette [*γδ* (*sacB-hyg***)***γδ*] to generate allelic exchange substrate for homologous recombination. Van911 restriction enzyme identifies a discontinuous palindrome interjected by five bases of sequence (CCAN_NNN^NTGG) to achieve a four-fragment ligation.

### Construction of specialized transducing phages

Shuttle phasmid phAE159 proliferates at 30 °C and does not propagate in the infected mycobacterial cell at 37 °C. Shuttle phasmid phAE159 accommodates up to 10 kb of recombinant DNA into mycobacterial cells. The mycobacteriophage carrying AES disrupts a precise gene in mycobacteria through deletion–substitution. The AES with hygromycin resistance marker differs from the phasmid, and the use of in vitro lambda packaging extract (Epicentre, USA) helps in increasing the efficiency of transformation and also in the selection of phasmid DNA. Then the phasmid DNA was electroporated into *M. smegmatis* to produce specialized transducing phages (STPs) at permissive temperature of 30 °C. These phages were further propagated using MP buffer (1 M Tris pH 8, 5 M NaCl, 1 M MgCl_2,_ 1 M CaCl_2_) to obtain high-titre allele-specific STPs.

### Specialized transduction

Specialized transduction was performed at 37 °C for the augmentation of STPs. In this temperature transfer of DNA to the host (mycobacterium) occurs with homologous recombination. The transduced *M. tuberculosis* cells were selected on hygromycin plate (150 μg/mL) after transduction. A minimum of five colonies appeared on the plate and was confirmed by PCR and Sanger sequencing using hyg as a forward primer and RHS as a reverse primer to confirm homologous recombination. For complementation of Rv0148, the gene was cloned into pMV261 vector harbouring *hsp*60 promoter. The construct was then electroporated into the ∆0148 strain and colonies were screened on the 7H10 plate with antibiotic selection (kanamycin 20 mg mL). Complementation was confirmed by PCR using Rv0148 primers and the complemented strain labelled as CΔ0148.

### In vitro growth kinetics of ∆0148

To check whether the disruption of Rv0148 fetched any changes in the in vitro growth of H_37_Rv, ∆0148 and CΔ0148 strains, log-phase cultures were grown in Middlebrook 7H9-OADC-Tween media and incubated at 37 °C with 180 rpm shaking. Aliquots of 100 μl culture were taken at different time points and OD values were measured at 600 nm using spectrophotometer (Eppendorf) on days 0, 3, 6, 9, 12 and 17. Survival of wild type, ∆0148 and CΔ0148 strains was analysed by plating the serial diluted broth cultures in 7H10 OADC agar without Tween-80. The plates were incubated for 3–4 weeks and the colony forming units were calculated.

### Colony morphology

The colony morphology of the mutant was determined by comparing with complementary strain and wild type. The strains were grown in liquid 7H9 media to mid-log phase and 10 μl of culture was inoculated to 7H10 agar plate supplemented with hygromycin. The plates were incubated at 37 °C for 3–4 weeks for colony formation.

### RNA extraction and qPCR

Bacterial culture 100 mL was grown in 7H9 media to mid-log phase and the growth was arrested in ice and centrifuged at 4 °C. The pellet was suspended in trizol (Invitrogen, USA). Cells were disrupted using 0.1 mm zirconium beads in a mini bead beater. The total RNA was extracted using the RNeasy purification kit (Qiagen, Germany) according to the manufacturer’s protocol and quantified using ND-1000 Nanodrop spectrophotometer (Nanodrop Technologies). It was stored at − 80 °C. To determine the relative mRNA through qPCR, the first strand cDNA was synthesized from 1 μg of RNA using Maxima first strand cDNA synthesis kit (Thermo Scientific, USA)**.** Quantitative qPCR primers for the Rv0148, *htdy* and 16S rRNA genes were designed (Table S2). qPCR was carried out using SYBR Green Master Mix (Thermo Scientific, USA) according to manufacturer’s instructions using the Applied Biosystems 7300 real-time PCR system. Control reactions for each sample were carried out in the absence of reverse transcriptase to look over for DNA contamination. The amplification conditions for all reactions were 1 cycle of 50 °C for 2 min, 95 °C for 10 min followed by 40 cycles of 95 °C for 15 s and 60 °C for 1 min**.** qPCR data analysis was carried out by relative quantification of Rv0148, *htdy* and CΔ0148 gene expression using comparative calculated threshold (CT) method. For each qPCR run, the CT cycle was normalized to the internal control 16S rRNA gene amplified from the corresponding sample. Statistical analysis was carried out using SDS 2.4 software and relative quantity (RQ value) method. The data presented are averages of three independent experiments.

### Infection of knockout strain in THP-1 cell lines

Intracellular viability of the knockout strain was determined using macrophage cell line infection studies. THP-1 cells were grown in RPMI media with 10% fetal bovine serum. Cells were further grown to reach 1 × 10^6^ cells, seeded onto 24-well plates and differentiated into macrophages using 50 mM phorbol 12-myristate 13-acetate. Plates were incubated at 37 °C in the presence of 5% CO_2_ incubator for 2 days, washed with RPMI medium and left overnight. Macrophages were then infected in triplicate with H_37_Rv, ∆0148 and CΔ0148 with multiplicity of infection of 1:10. Phagocytosis was allowed to take place for 4 h, after which the infected macrophages were incubated with RPMI containing streptomycin to eliminate extracellular bacteria. Further, the cell-free culture supernatants were collected on days 0, 1, 3, 5 and 7 post-infection and stored at − 80 °C for cytokine analysis.

### Elisa

The supernatants derived from THP-1 cell lines infected with H_37_Rv, ∆0148 and CΔ0148 at different intervals were evaluated for expression of cytokines IL-4, IL-6, IL-10, IL-1β, TNF, IL-2, INF-γ, IL-12 (p40) and IL-12 (p70) by sandwich ELISA using BD ELISA kit (BD pharmingen, USA) according to manufacturer’s protocol.

### Role of knockout mutant during oxidative stress

To understand the role of oxidative and nitrogen stress in H_37_Rv, ∆0148 and CΔ0148, the strains were grown to mid-log phase in 7H9-OADC-T medium and exposed to various stress components such as electron transport chain (ETC) inhibitors, peroxides, sulphoxides, thiol stress with slight modification to the procedure by [27] and OD values were measured at 600 nm (OD600) for 4, 24 and 48 h time points. The graphs were plotted and significance was reported considering *P* < 0.01 and *P* < 0.001.

### MGIT and drug susceptibility testing

The H_37_Rv and knockout strains were grown on LJ solid media, 0.5 McFarland culture suspension was used for MGIT inoculation and drug susceptibility testing (DST) was performed for all the culture-positive MGIT tubes after 4 days. The growth control (GC) was prepared by diluting the cultures in the ratio of 1:100. The test cultures were prepared using saline in 1:5 dilution; 800 μL of supplement was added to tubes according to manufacturer's protocol; 100µL streptomycin 1 μg mL, isoniazid 0.1 μg mL, rifampicin 1 μg mL and ethambutol 5 μg mL (SIRE kit, BD). Kanamycin 2.5 μg mL, ofloxacin 2 μg mL and amikacin 1 μg mL was added to the respective tubes; and finally 500 μL of culture was added to all the tubes and loaded in BACTEC MGIT 960. Tubes were recapped, mixed well and fixed into the BACTEC MGIT 960 using the antimicrobial susceptibility testing set entry protocol. BACTEC MGIT 960 instrument monitored susceptibility test until the growth unit of the GC reaches 400 [38].

### Interactome analysis to predict drug-resistance partners

To predict the interacting genes involved in drug resistance, STITCH database was used and further analysed using a bioinformatics tool STRING.

### Expression of *htdy* in the presence and absence of Rv0148

H_37_Rv, mutant and complimentary strains were grown in 7H9 media to log phase according to the growth conditions mentioned earlier and RNA was extracted. Individual and co-expression of *htdy* were analysed through qPCR in mutant and complementary strains and H_37_Rv. The Ct and *R* values were further considered for analysing the folds of gene expression.

### Statistical analysis

The data represented all the three independent experiments carried out using replicates. Mean, standard error mean (SEM) and Two-way ANOVA was used to analyse the data obtained from in vitro kinetics, ELISA and oxidative stress assays (**P < 0.01 was said to be significant and ***P < 0.001 was said to be highly significant). Real time PCR data was analysed using SDS software and relative quantity method.

## Supplementary information

**Additional file 1: Table S1.** HMM analysis files showing 1270 significant sequences from Swissprot database.

**Additional file 2: Table S2.** List of primers used in this study. **Table S3.** Data showing CT values for confirmation of gene knockout using real time PCR. **Table S4.** Data showing the CT values of *htdy* expression in gene knockout mutant.

**Additional file 3: Figure S1.** Restriction enzyme digestion of Rv0148 &Rv3389 and cloning. **(A)** Confirmation of recombinant clones in pGEX4T-1 M: 1 kb marker, Lane 1 to 4; pGEX4T-1 as control with 4.9 kb size, Lane 5to 8 recombinant clones, Lane 8 showing insert size 858 bp (**B)** Confirmation of recombinant clones using restriction digestion in pRSET-B M; marker, Lane 1; Control pRSET-B correlating 2.8 kb, Lane 2 to 6 showing recombinant clones with insert size 969 bp.

**Additional file 4: Figure S2.** Construction of knockout. **(A)** allelic exchange substrate construction M: 1 kb Marker, Lane 1: SacB digested with van911 showing four fragments 3.6 kb, 1.6 kb, 979 bp, 567 bp, Lane 2& 3: 0148 AES construct showing four fragments 3.6 kb, 1.6 kb, 837 RA, 605LA. **(B)** Packaging of AES with phAE159. M: 1 kb ladder, Lane 1: phAE159 digested with pac-I showing 50 kb and insert 3.8 kb, Lane 2& 3 clones without insert, Lane 4& 5 clone digested with pac-I showing phAE159 50 kb and 6.6 kb AES **(C)** Confirmation of knockout using PCR M: 1 kb ladder, Lane 1: Rv DNA amplified with right arm, Lane 2,4,5: knockout DNA amplified with hyg Forward primer and right arm reverse primer, Lane 6: Rv DNA not showed amplification with hyg & reverse primer**.**

**Additional file 5: Figure S3.** Intersection of genes and role in drug resistance. Intersection of Rv0148, Htdy and EIS with the three drugs kanamycin, amikacin and streptomycin resulting in 57 interacting proteins.

## Data Availability

All data generated or analyzed during this study are included in this published article and its supplementary information files.

## Abbreviations

SDRs: Short-chain dehydrogenases/reductases
TB: Tuberculosis
*M. tuberculosis*: *Mycobacterium tuberculosis*
RNS: Reactive nitrogen species
ROS: Reactive oxygen species
AhpC: Alkylhydroperoxidase reductase
AhpE: Peroxiredoxin
Tpx: Thioredoxin reductase
MIC: Minimum inhibitory concentration
STPKs: Serine-threonine protein kinases
NAD: Nicotinamide adenine dinucleotide
PDB: Protein database
RT-PCR: Real-time Polymerase chain reaction
ELISA: Enzyme-linked immunosorbent assay
MGIT: Mycobacteria growth indicator tube
