# Supplementary figures and images for: Protein–protein interaction of Rv0148 with Htdy and its predicted role towards drug resistance in Mycobacterium tuberculosis
Source: BMC Microbiol. 2020 Apr 15;20:93. doi: 10.1186/s12866-020-01763-1 (PMC7161113; doi:10.1186/s12866-020-01763-1)

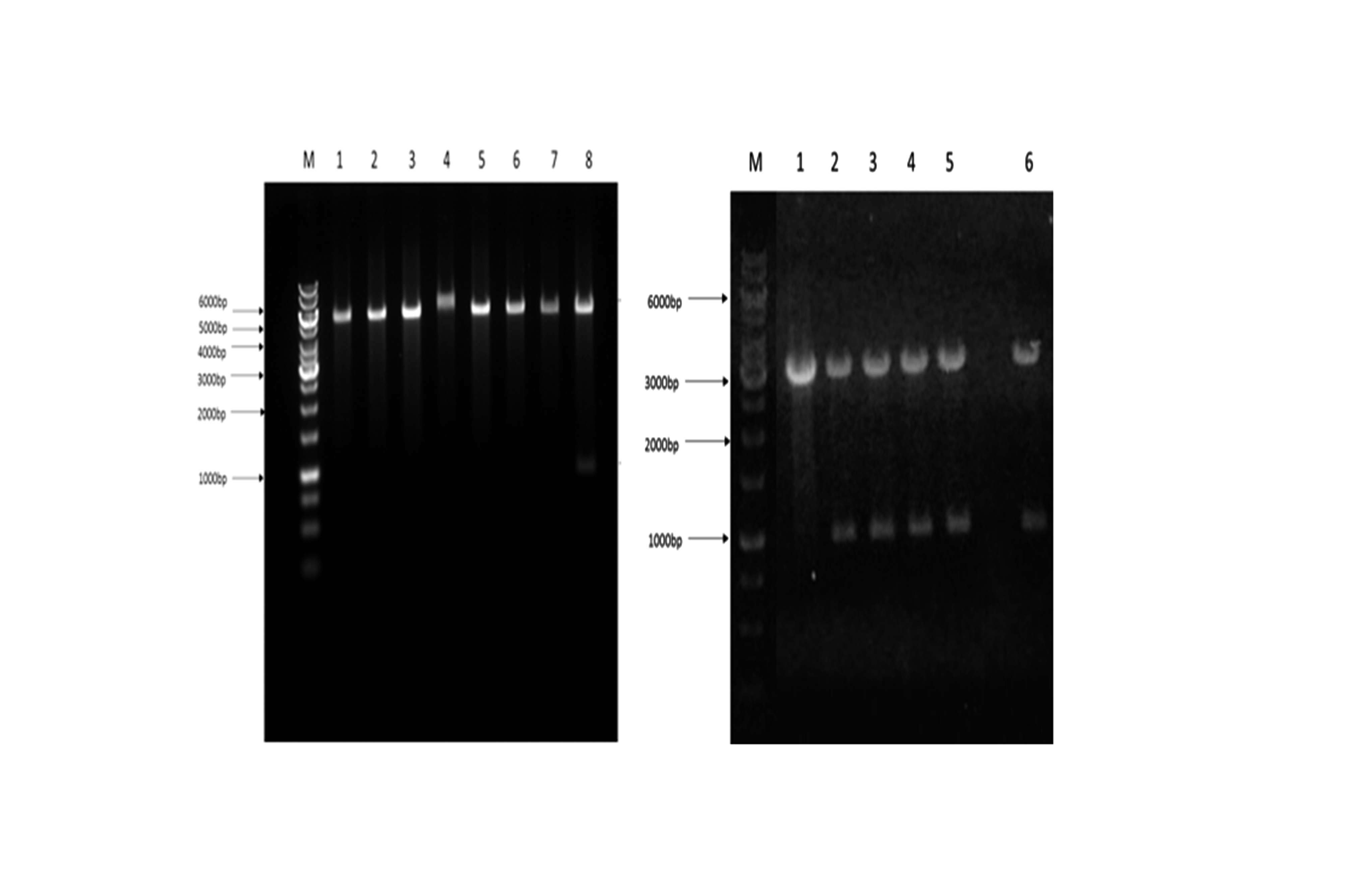

Supplement: Supplementary file 3 — Additional file 3: Figure S1. Restriction enzyme digestion of Rv0148 &Rv3389 and cloning. (A) Confirmation of recombinant clones in pGEX4T-1 M: 1 kb marker, Lane 1 to 4; pGEX4T-1 as control with 4.9 kb size, Lane 5to 8 recombinant clones, Lane 8 showing insert size 858 bp (B) Confirmation of recombinant clones using restriction digestion in pRSET-B M; marker, Lane 1; Control pRSET-B correlating 2.8 kb, Lane 2 to 6 showing recombinant clones with insert size 969 bp. [file 12866_2020_1763_MOESM3_ESM.tif]

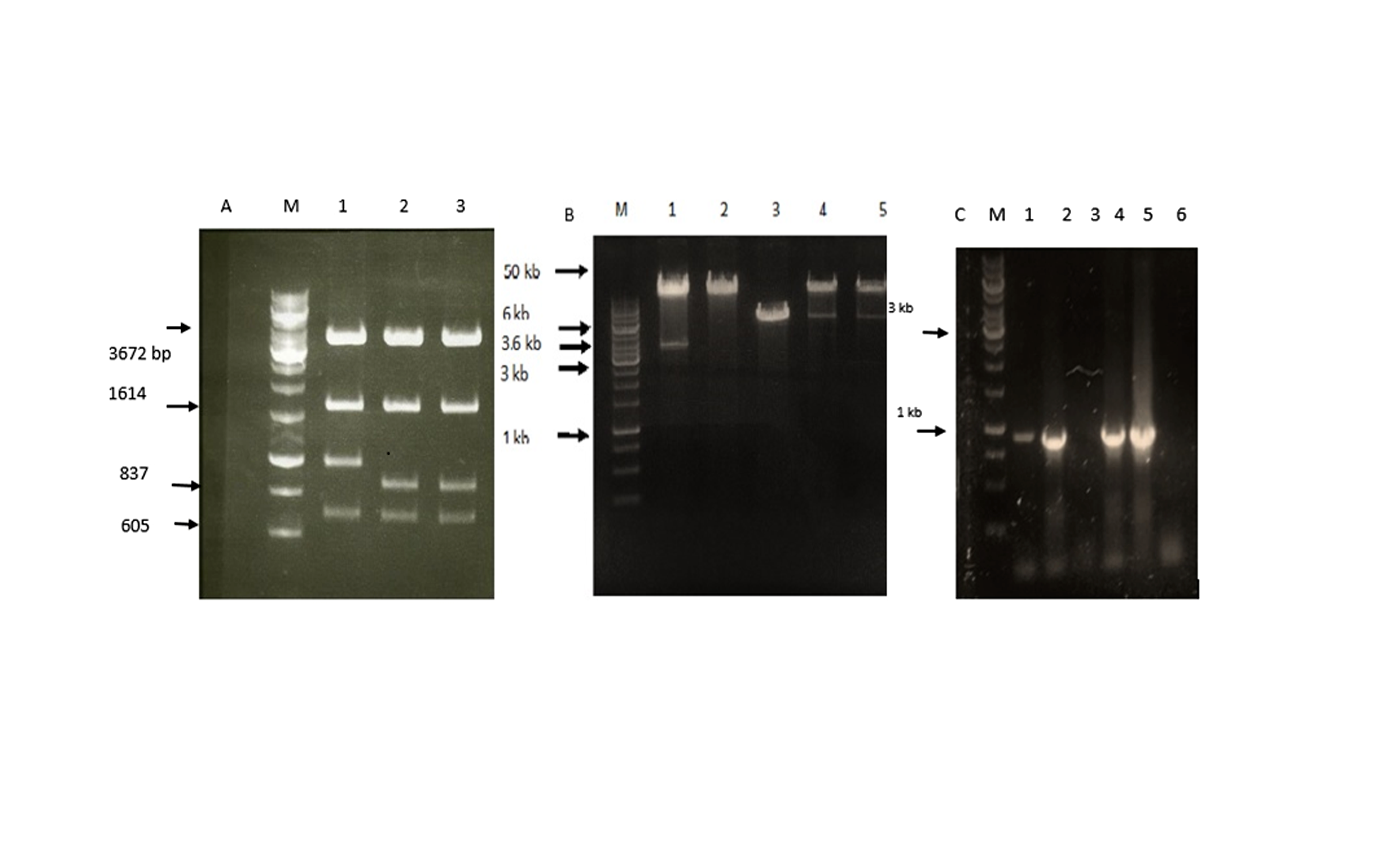

Supplement: Supplementary file 4 — Additional file 4: Figure S2. Construction of knockout. (A) allelic exchange substrate construction M: 1 kb Marker, Lane 1: SacB digested with van911 showing four fragments 3.6 kb, 1.6 kb, 979 bp, 567 bp, Lane 2& 3: 0148 AES construct showing four fragments 3.6 kb, 1.6 kb, 837 RA, 605LA. (B) Packaging of AES with phAE159. M: 1 kb ladder, Lane 1: phAE159 digested with pac-I showing 50 kb and insert 3.8 kb, Lane 2& 3 clones without insert, Lane 4& 5 clone digested with pac-I showing phAE159 50 kb and 6.6 kb AES (C) Confirmation of knockout using PCR M: 1 kb ladder, Lane 1: Rv DNA amplified with right arm, Lane 2,4,5: knockout DNA amplified with hyg Forward primer and right arm reverse primer, Lane 6: Rv DNA not showed amplification with hyg & reverse primer. [file 12866_2020_1763_MOESM4_ESM.tif]

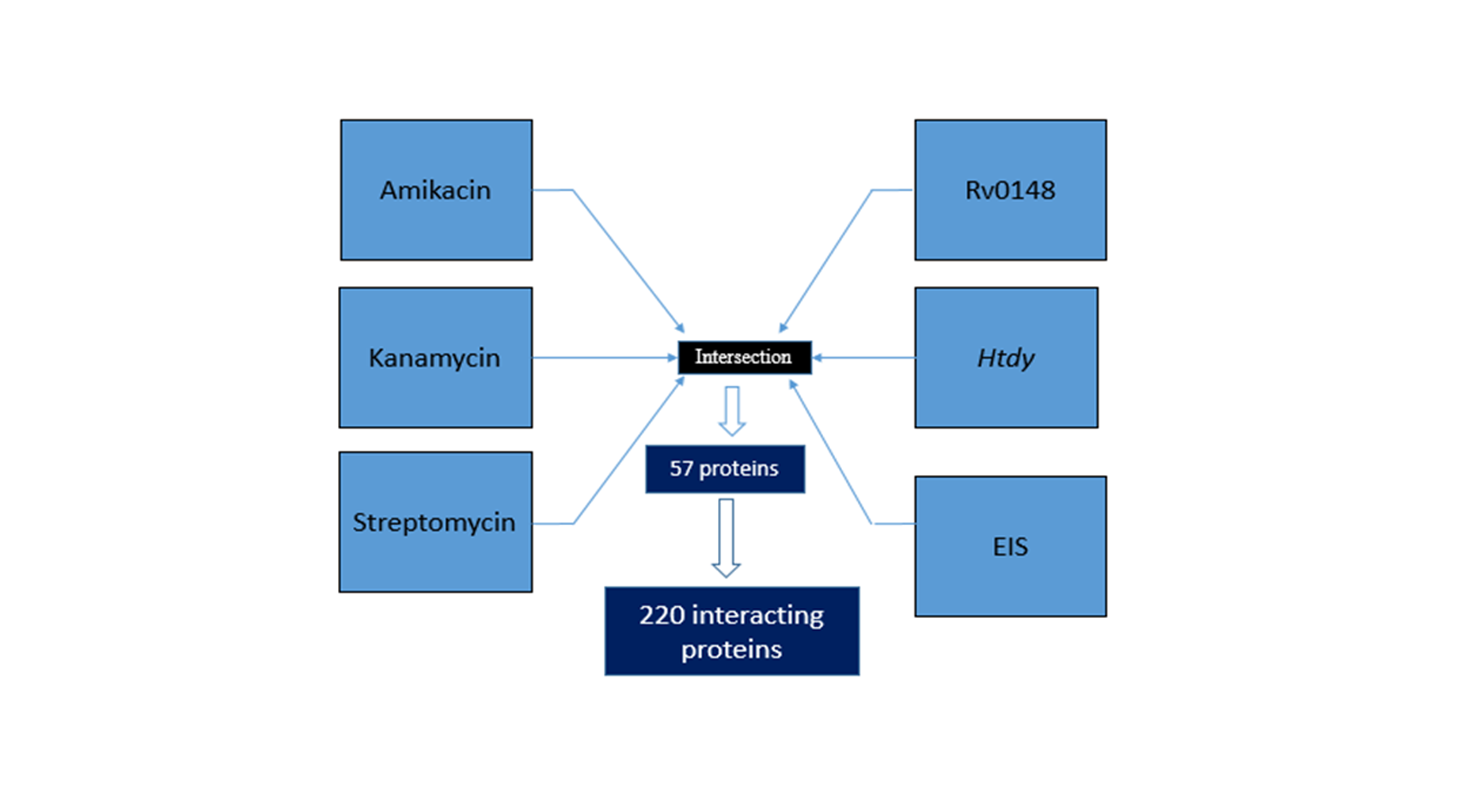

Supplement: Supplementary file 5 — Additional file 5: Figure S3. Intersection of genes and role in drug resistance. Intersection of Rv0148, Htdy and EIS with the three drugs kanamycin, amikacin and streptomycin resulting in 57 interacting proteins. [file 12866_2020_1763_MOESM5_ESM.tif]
